# Variability in the serial interval of COVID-19 in South Korea: a comprehensive analysis of age and regional influences

**DOI:** 10.3389/fpubh.2024.1362909

**Published:** 2024-03-07

**Authors:** Hyosun Lee, Gira Lee, Tobhin Kim, Suhyeon Kim, Hyoeun Kim, Sunmi Lee

**Affiliations:** ^1^Department of Applied Mathematics, Kyung Hee University, Yongin, Republic of Korea; ^2^Humanitas College, Kyung Hee University, Seoul, Republic of Korea

**Keywords:** COVID-19, infector-infectee pairs, age-specific and region-specific serial interval, time-varying reproduction number, various interventions

## Abstract

**Introduction:**

Quantifying the transmissibility over time, particularly by region and age, using parameters such as serial interval and time-varying reproduction number, helps in formulating targeted interventions. Moreover, considering the impact of geographical factors on transmission provides valuable insights into the effectiveness of control measures.

**Methods:**

Drawing on a comprehensive dataset of COVID-19 cases in South Korea, we analyzed transmission dynamics with a focus on age and regional variations. The dataset, compiled through the efforts of dedicated epidemiologists, includes information on symptom onset dates, enabling detailed investigations. The pandemic was divided into distinct phases, aligning with changes in policies, emergence of variants, and vaccination efforts. We analyzed various interventions such as social distancing, vaccination rates, school closures, and population density. Key parameters like serial interval, heatmaps, and time-varying reproduction numbers were used to quantify age and region-specific transmission trends.

**Results:**

Analysis of transmission pairs within age groups highlighted the significant impact of school closure policies on the spread among individuals aged 0-19. This analysis also shed light on transmission dynamics within familial and educational settings. Changes in confirmed cases over time revealed a decrease in spread among individuals aged 65 and older, attributed to higher vaccination rates. Conversely, densely populated metropolitan areas experienced an increase in confirmed cases. Examination of time-varying reproduction numbers by region uncovered heterogeneity in transmission patterns, with regions implementing strict social distancing measures showing both increased confirmed cases and delayed spread, indicating the effectiveness of these policies.

**Discussion:**

Our findings underscore the importance of evaluating and tailoring epidemic control policies based on key COVID-19 parameters. The analysis of social distancing measures, school closures, and vaccine impact provides valuable insights into controlling transmission. By quantifying the impact of these interventions on different age groups and regions, we contribute to the ongoing efforts to combat the COVID-19 pandemic effectively.

## 1 Introduction

As the Coronavirus Disease (COVID-19) began to rapidly spread, the World Health Organization (WHO) declared it a global pandemic in 2020 (1). This declaration prompted governments around the world to introduce diverse interventions aimed at curbing the transmission of this infectious disease and minimizing its impact on public health. Even in the early stages of an epidemic, it is essential to identify behavioral patterns by age and region over time. This understanding is fundamental for ensuring effective control and preventing further transmission. The evaluation of each intervention, grounded in essential epidemiological parameters and data analysis, is a critical step in gauging the efficacy of public health measures and non-pharmaceutical interventions.

The serial interval, which denotes the duration between the onset of symptoms in an infector and a subsequent infectee, is a significant parameter. This parameter, derived from available data, can serve as a versatile indicator, particularly in assessing the infection's spread. On the other hand, the generation interval represents the period between infection in an infector and infection in an infectee (2). While the generation interval is a crucial metric for comprehending transmission dynamics, it can be challenging to ascertain due to data constraints. Consequently, the serial interval is often estimated and employed as a proxy for the generation interval, which offers the advantage of more accessible data collection (3). This substitution enables us to gain insights into infection patterns over time, across different age and regional groups.

Numerous studies have highlighted the valuable role of data-derived serial intervals in shaping effective policy implementation. Li et al. (4), Du et al. (5), Zhao et al. (6), and Wang et al. (7) demonstrated how they estimate optimal distributions by fitting serial intervals to various statistical distributions. Meanwhile, Yang et al. (8) and Aghaali et al. (9) employed contact tracing data to estimate serial interval distributions. Knight et al. (10) and Najafi et al. (11) harnessed serial intervals to illustrate the time-varying reproduction number $R_{t}$, providing valuable insights into the patterns of disease spread. Clearly, the serial interval has proven instrumental in assessing transmissibility over time.

In prior investigations, the serial interval has served as a valuable metric for examining the transmission dynamics across age and region. Ali et al. (12) highlighted that the serial interval of COVID-19 becomes longer when isolation is delayed but contracts due to non-pharmaceutical interventions. They delved into variations in the distribution of the serial interval concerning the transmission of COVID-19 in Hong Kong, shedding light on the disease's dissemination patterns and the efficacy of control strategies. In another study, Kim et al. (13) reported an average serial interval of 3.78 days, with briefer intervals for children (3.0 days) and prolonged intervals for adults (5.0 days). These findings underscore the importance of enforcing stringent public health measures across all age groups, especially among children.

Our main aim is to investigate the heterogeneity of COVID-19's serial interval in South Korea, with a focus on age and region as key factors. In South Korea, we were fortunate to have access to valuable data collected through the dedicated efforts of epidemiologists and transparent public health policies, which greatly facilitated our analysis. This dataset covers the period from January 2020 to December 2021. We divide this timeline into four distinct phases. Each period is categorized considering various trends in the COVID-19 pandemic, including suppression policies, the emergence of variants, changes in transmission dynamics, and the introduction of vaccines. More details can be found in Ha et al. (14).

Our analysis goes beyond investigating how these serial intervals differ with respect to age and region over time. It also examines how various interventions influence serial intervals. Despite the considerable body of research on COVID-19, there's a noticeable gap when it comes to age- and region-specific investigations. This gap makes it challenging to gain a clear understanding of how population demographics and regional contexts impact the transmission of infectious diseases. Hence, this study delves into the serial interval in the context of age and region. Furthermore, in our efforts to evaluate transmissibility across regions, we compute the time-varying reproduction number ($R_{t}$) using the most appropriate region-specific serial interval. Our goal is to shed light on the disease's transmission patterns and the effectiveness of response strategies. This study endeavors to provide a distinct perspective and analytical approach compared to previous research, with the aim of contributing to the development of policies for managing infectious diseases.

## 2 Materials and methods

### 2.1 Data

Epidemiological data from January 2020 to December 2021 is provided by the Korea Disease Control and Prevention Agency (KDCA) (15). We utilized data from 72,423 transmission pairs, including symptom onset dates, for our research. The KDCA facilitated data collection by initiating contact tracing and maintaining records thereof. Starting from February 24, 2020, a comprehensive investigation via contact tracing was conducted, with the COVID-19 epidemiological investigation support system (K-EISS) being employed from March 1, 2020 onwards. During this period, epidemiological investigation teams in various cities and provinces conducted retrospective investigations and gathered information on both infectors and infectees through telephone or text surveys. Additionally, in instances of significant outbreaks with a high number of confirmed cases, epidemiological investigators from the KDCA were deployed to gather information on these cases. Furthermore, starting from June 10, 2020, the electronic entry and exit registration system (QR code) was introduced to streamline contact tracing efforts by efficiently recording the movement history of all individuals. These policies and methodologies are documented and can be referenced in the KDCA (16, 17).

Through this, we analyzed nationwide heterogeneity using the serial interval. In our study, we categorized age, region, and time into four, seven, and four groups, respectively. Categorized into four distinct age groups—1 (0–19), 2 (20–29), 3 (30–64), and 4 (≥65). Classified into seven regional groups—1 (Metro: Seoul, Gyeonggi, Incheon), 2 (Gyeongnam+: Gyeongnam, Busan, Ulsan), 3 (Gyeongbuk+: Gyeongbuk, Daegu), 4 (Jeonlla+: Jeonbuk, Jeonnam, Gwangju), 5 (Chungcheong+: Chungbuk, Chungnam, Daejeon, Sejong), 6 (Gangwon), and 7 (Jeju). Divided across four specific periods—Period 1 (P1): January 19 to August 11, Period 2 (P2): August 12 to November 12, 2020, Period 3 (P3): November 13, 2020, to July 6, 2021, Period 4 (P4): July 7 to December 31, 2021. The primary factors under consideration include age, region, and symptom onset date (14).

- Period 1 (P1): notable outbreaks occurred in specific religious gatherings, such as the Shincheonji Church in Daegu. Social distancing measures and enhanced preventive actions were implemented in response.
- Period 2 (P2): the outbreak predominantly affected the metropolitan area. Large-scale events and various multi-use facilities, along with the rapid spread in the capital region, led to prioritized reinforcement of epidemic control measures.
- Period 3 (P3): the emergence of major variant viruses, widespread transmission across the nation, and an increase in infections in daily life settings prompted diverse interventions, including social distancing measures and targeted testing.
- Period 4 (P4): the Delta variant's spread, group infections in various facilities, an elevated proportion of foreign cases, the introduction of the Omicron variant, and subsequent intensified social distancing measures and comprehensive epidemic responses marked this period.

**Figure 1:**
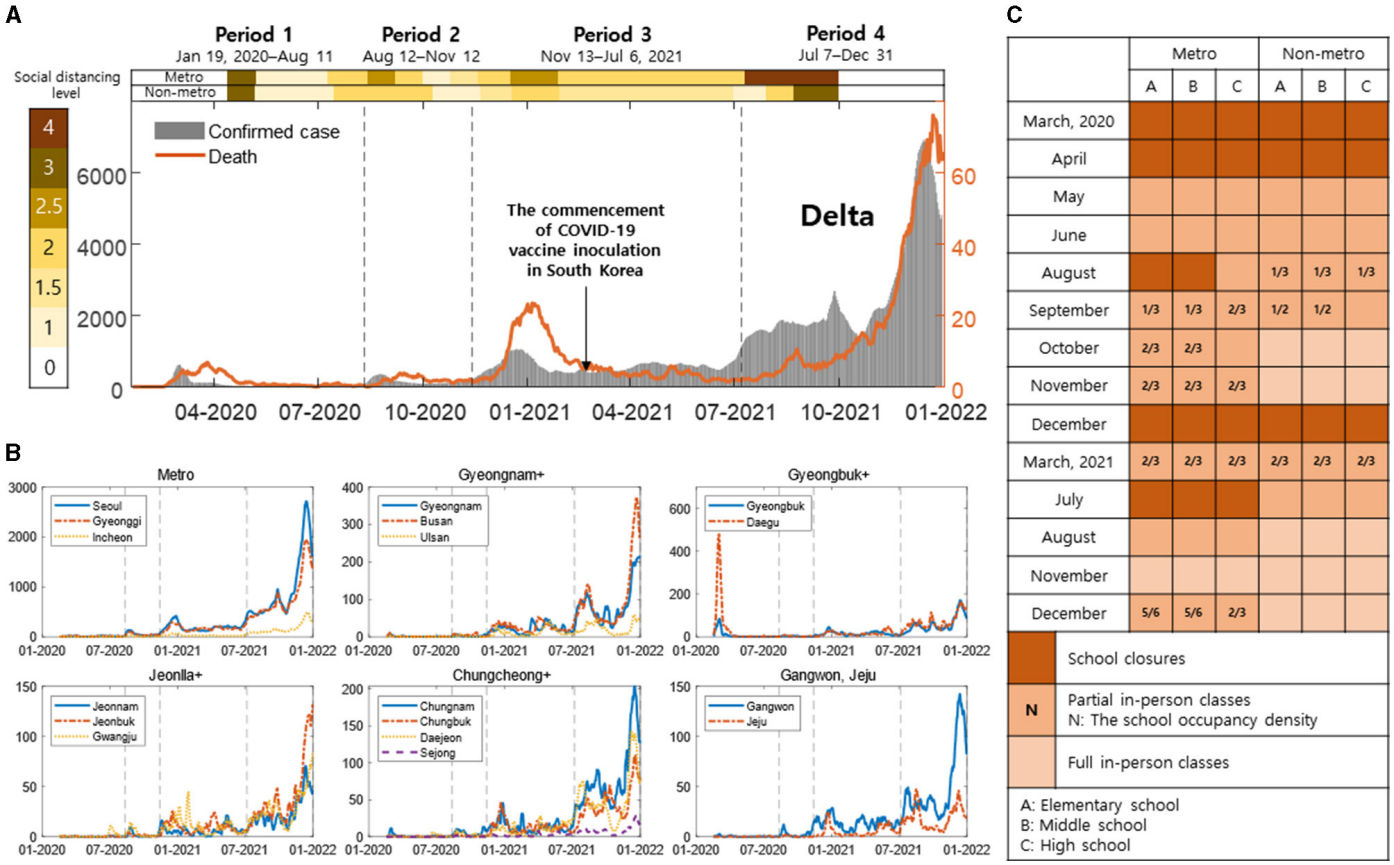
**(A)** Daily confirmed cases, deaths, vaccines, variants, and social distancing measures are shown [the gray bars: daily confirmed cases (left *y*-axis), the orange line: daily deaths (right *y*-axis)]. The intensity of color in the background indicates the level of social distancing, getting darker as it increases. **(B)** Region-specific daily confirmed cases are shown with the black dashed line indicating Period 1–4. **(C)** This shows the school closure policies in metropolitan and non-metropolitan areas. **(A–C)** Represent elementary, middle, and high schools, respectively. The intensity of color represents full, partial in-person classes, and school closures, with N indicating density in partial school closures.

Figure 1 presents a comprehensive overview of the COVID-19 situation in South Korea spanning from January 2020 to December 2021. Figure 1A shows daily confirmed cases, deaths, vaccines, variants, and social distancing level in South Korea. The gray bar shows daily confirmed cases (left *y*-axis), and the orange line shows daily deaths (right *y*-axis). Social distancing levels are divided into metropolitan (metro) and non-metropolitan (non-metro), and the darker the color, the higher it is. Vaccination in high-risk groups began, and vaccinations nationwide began on February 26, 2021. Subsequently, vaccinations for those aged 18 and below commenced on June 27, 2021. In Period 4, it indicates the dominance of the Delta variant.

Figure 1B represents region-specific daily confirmed cases, with the black dashed line indicating Period 1–4. In Metro, Seoul, Gyeonggi, and Incheon are shown with blue lines, and different styles of red and yellow dashed lines. In Gyeongnam+, Gyeongnam, Busan, and Ulsan are depicted with blue lines, and different styles of red and yellow dashed lines. In Gyeongbuk+, Gyeongbuk, and Daegu are shown with blue and red dashed lines, respectively. In Jeonlla+, Jeonnam, Jeonbuk, and Gwangju are illustrated with blue lines, and different styles of red and yellow dashed lines. In Chungcheong+, Chungnam, Chungbuk, Daejeon, and Sejong are represented with blue lines, and different styles of red, yellow, and purple dashed lines. Gangwon and Jeju are shown with blue and red dashed lines, respectively.

Figure 1C depicts the in-person class policy for elementary, middle, and high schools in South Korea, as announced by the Ministry of Education. Figurea 1A–C respectively represent elementary, middle, and high schools. It is presented divided into metropolitan (metro) and non-metropolitan (non-metro), with the intensity of color indicating full, partial in-person classes, and school closures. In partial no in-person classes, N represents density. Table 1 shows the number of infector-infectee pairs by age and regional groups for periods 1 to 4.

**Table 1:** The number of transmission pairs is represented by age and regional groups over given periods.

	**Period 1**	**Period 2**	**Period 3**	**Period 4**
0–19	73	136	2,324	11,941
20–29	242	302	3,480	4,927
30–64	902	1,729	17,851	19,157
≥65	290	597	3,580	4,892
Metro	852	2,071	18,859	16,844
Gyeongnam+	71	131	2,892	8,747
Gyeongbuk+	337	55	1,746	4,412
Jeonlla+	47	110	1,560	4,949
Chungcheong+	150	243	1,315	4,867
Gangwon	16	127	377	447
Jeju	1	7	352	578

### 2.2 Estimation of serial interval and time-varying reproduction number

$\left(O_{j}^{i},E_{j}^{i}\right)$ is the observed symptom onset date of the infector and infectee forming the transmission pair in the *j*-th period in the *i*-th age or regional group. $S_{j}^{i}$ represents the serial interval corresponding to the *j*-th period in the *i*-th age or regional group as follows:


(1) $$\begin{array}{l} s_{j}^{i}=e_{j}^{i}-o_{j}^{i} \end{array}$$

where $\left(s_{j}^{i},o_{j}^{i},e_{j}^{i}\right)$ is the serial interval and symptom onset date of the infector and infectee, respectively, in the *j*-th period in the *i*-th age or regional group included in ($S_{j}^{i},O_{j}^{i},E_{j}^{i}$). Through Equation (1), we derive the serial interval from contact tracing data. $f_{j}^{i}\left(\cdot \right)$ represents the probability density function (PDF) of the serial interval of the *j*-th period in the *i*-th age or regional group. The log likelihood to obtain the distribution of the serial interval corresponding to the *j*-th period in the *i*-th age or regional group is as follows:


(2) $$l_{j}^{i}(\alpha_{j}^{i},\beta_{j}^{i}|E_{j}^{i},O_{j}^{i})=\overset{N_{j}^{i}}{\Sigma }logf_{j}^{i}(e_{j}^{i},o_{j}^{i}|\alpha_{j}^{i},\beta_{j}^{i})$$

where $N_{j}^{i}$ is the number of transmission pair corresponding to the *j*-th period in the *i*-th age or regional group and $\alpha_{j}^{i},\beta_{j}^{i}$ are the two parameters of the PDF $f_{j}^{i}$. We estimate the distribution of PDF $f_{j}^{i}\left(\cdot \right)$ for normal, lognormal, gamma, and Weibull distributions through maximum likelihood estimation (MLE) with Equation (2) (2, 18). It is crucial for understanding the spread speed and patterns of infectious diseases. It helps us determine how quickly an infected individual transmits the disease to others, playing a vital role in predicting the spread of the disease and formulating response plans. Therefore, the mean serial interval is of utmost importance in infectious disease modeling and prediction. We extract the serial interval from the data based on the symptom onset dates of infectors and infectees.

AIC (Akaike Information Criterion) is one of the useful statistical criteria for model selection and evaluation. AIC is used to strike a balance between the goodness of fit of a model and its complexity. It aids in assessing model complexity and evaluating goodness of fit, helping in the selection of the most appropriate model. The basic formula is as follows:


(3) $$\begin{array}{l} AIC=-2log\left(likelihood\right)+2k \end{array}$$

where *k* represents the number of model parameters (19). AIC takes into consideration how well a model explains the data (lower likelihood values correspond to higher AIC) and how simple the model is (models with fewer parameters are more likely to have lower AIC). The model with the lowest AIC is considered the most suitable for the given data. We identify the most suitable distribution for the serial interval from the distribution with Equation (3), considering age and regional groups over time.

Time-dependent reproduction number (R(t)) is an important concept in epidemiology, indicating how an infectious disease changes over time. This concept provides crucial information about the spread patterns and control of the disease. We define the time-dependent reproduction number in the *i*-th regional group,


(4) $$\begin{array}{l} R^{i}\left(t\right)=\frac{I^{i}\left(t\right)}{\Lambda^{i}\left(t\right)} \end{array}$$

where the ratio of the number of new infected cases at time *t*, *I*^*i*^(*t*), and the total infection potential across all infected individuals at time *t*, Λ^*i*^(*t*) = *I*^*i*^(*t*−τ)·*s*^*i*^(τ) in the *i*-th regional group, if *s*(·) is the distribution of serial interval (3). According to the Equation (4), tracking R(t) helps understand how the rate of disease spread changes over time and assists in evaluating the effectiveness of control measures. It serves as a crucial tool in epidemiological research and pandemic response planning, especially aiding in monitoring the spread of infectious diseases and timely adjustment of control measures. If R(t) is greater than 1, the epidemic continues to spread, and if it is less than 1, the epidemic tends to decrease. We utilize the previously determined optimal distribution of the serial interval to estimate the regional $R^{i}\left(t\right)$.

## 3 Results

**Figure 2:**
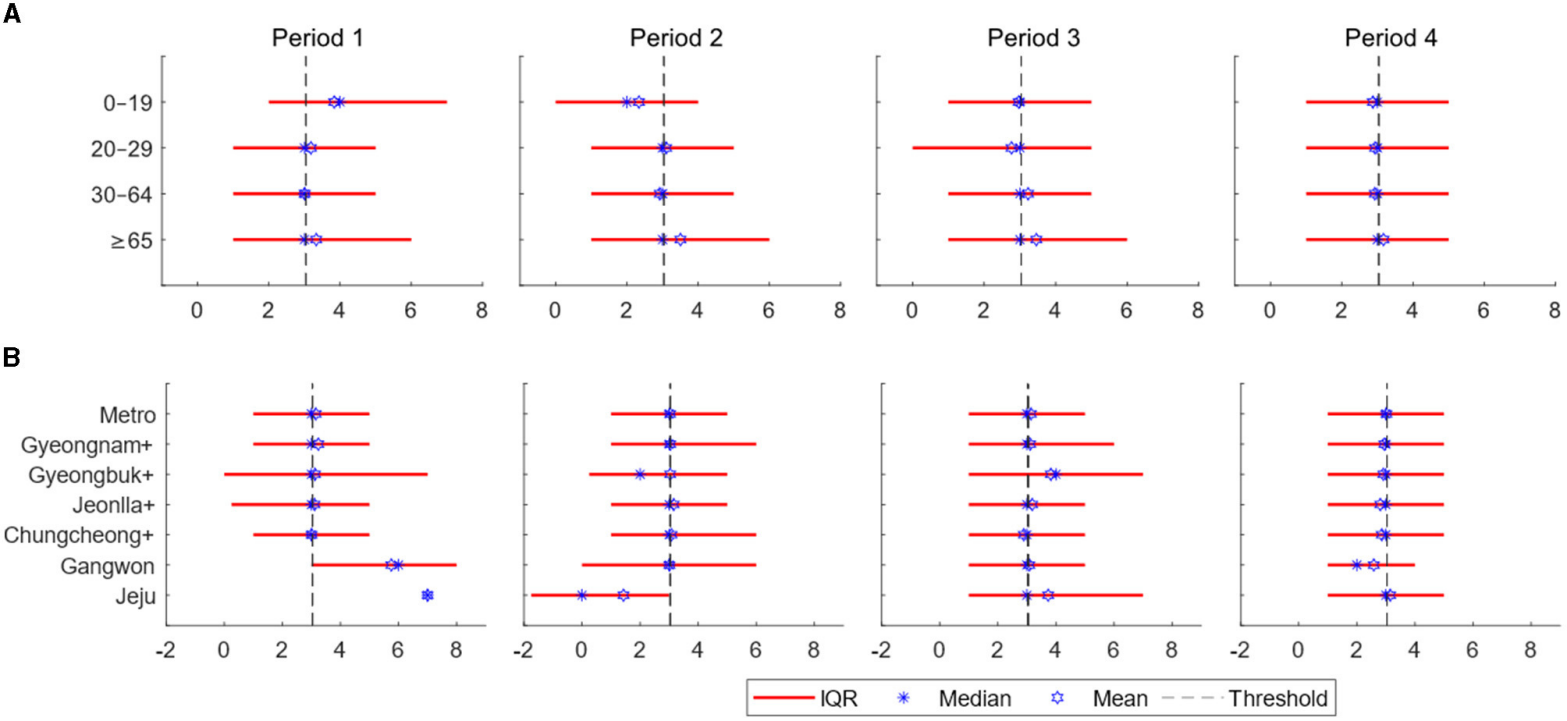
**(A)** Estimates of the serial interval are displayed over time according to age groups. **(B)** Estimates of the serial interval are displayed over time based on regions. The *x*-axis represents days, while the *y*-axis represents the regions [the interquartile range: red line, threshold (total mean): dashed black line, the mean for each age or regional group in the respective periods: blue star, and the median for each age or regional group in the respective periods: blue hexagram].

In this section, to understand the heterogeneity in the spread patterns, we presented the distribution of serial intervals, the number of infector-infectee pairs, demographic confirmed cases, vaccination rates, and time-varying reproduction numbers over time by age and regional groups using contact tracing data. Figure 2 displays bar plots illustrating age-specific serial intervals over time (Figure 2A) and region-specific serial intervals over time (Figure 2B). The *x*-axis represents the number of days, while the *y*-axis represents different age and regional groups. The red lines indicate the interquartile range (from the 25th to the 75th percentile) of the serial interval. The dashed black line represents the overall average of 3.0 across all time periods and groups. Stars indicate the median serial interval for each group in the corresponding periods, while hexagrams represent the mean serial interval for each group in their respective periods.

Table 2 represents the proportion of negative serial intervals in age and regional groups. Values with less than 30 data points are indicated with -. The overall proportion of negative serial interval is 13.0%. Across age groups, the range of negative serial interval proportions in Periods 1–4 is 9.6–16.2%, 12.6–20.6%, 13.8–16.7%, and 10.9–12.6%, respectively. Based on regional groups, the range in Periods 1–4 is 8.5–21.1%, 10.9–17.3%, 12.9–15.1%, and 10.4–12.9%. Over time, there is a trend of all age and regional groups converging toward the overall mean.

**Table 2:** The proportion of the negative serial interval for the respective age and regional groups.

	**Period 1**	**Period 2**	**Period 3**	**Period 4**
0–19	9.6%	20.6%	15.4%	10.9%
20–29	10.7%	12.6%	16.7%	11.4%
30–64	14.6%	15.4%	13.8%	12.4%
≥65	16.2%	13.2%	15.3%	12.6%
Metro	12.1%	15.2%	14.5%	10.8%
Gyeongnam+	8.5%	13.0%	15.1%	12.9%
Gyeongbuk+	21.1%	10.9%	12.9%	12.9%
Jeonlla+	12.8%	10.9%	15.1%	12.0%
Chungcheong+	14.7%	14.4%	14.6%	12.8%
Gangwon	-	17.3%	13.8%	12.3%
Jeju	-	-	13.6%	10.4%

**Figure 3:**
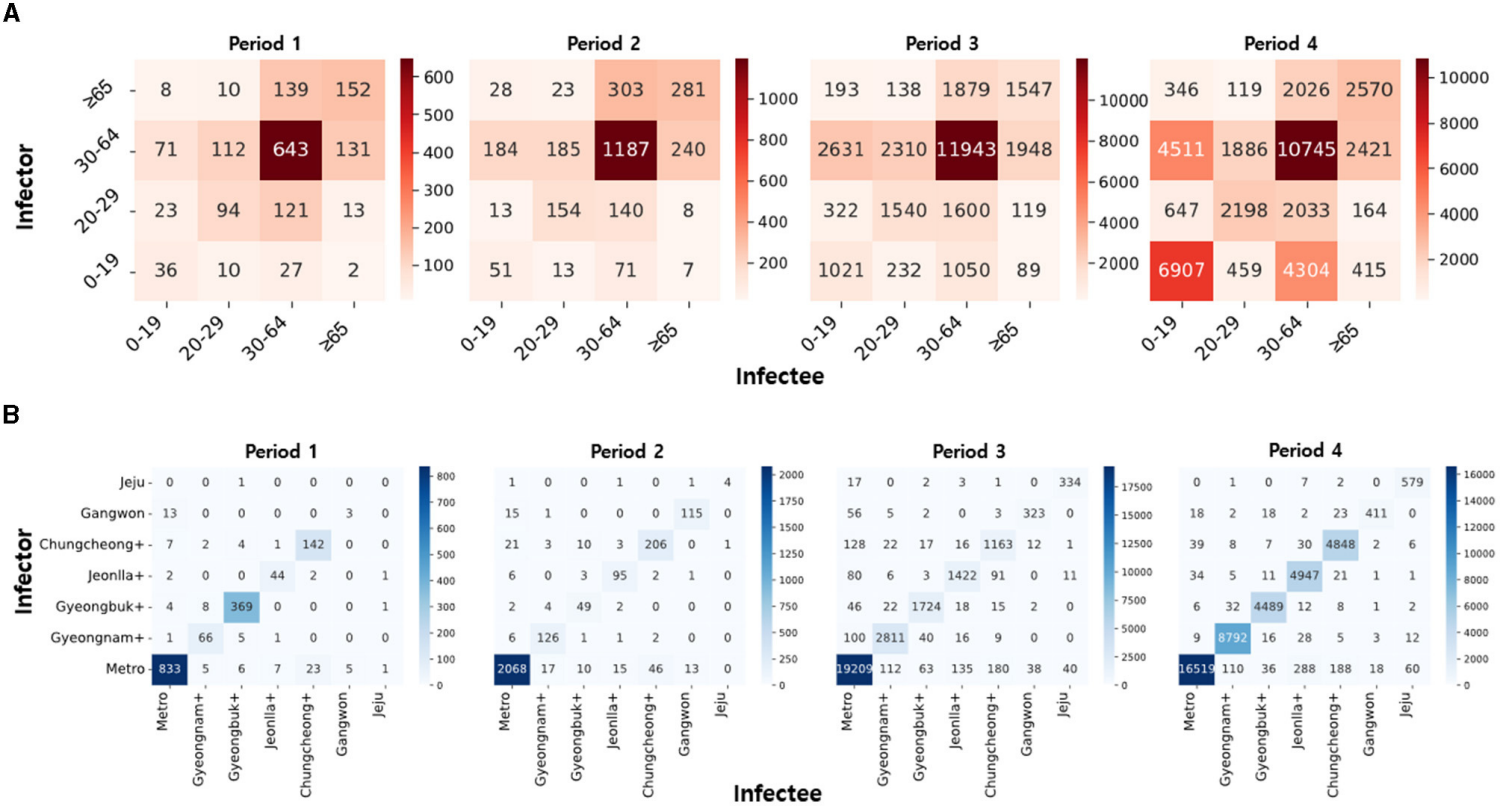
**(A)** The heatmaps of infector-infectee pairs are displayed over time based on age groups. **(B)** The heatmaps of infector-infectee pairs are displayed over time based on regions. Darker colors indicate a relatively higher count of pairs for the given time period.

Figure 3 presents the number of infector-infectee pairs across different age groups (Figure 3A) and regional groups (Figure 3B) for each period. The proportion of diagonal elements within the age group is high, with a notable prevalence of the 30–64 age group across all periods. This indicates active transmission within the demographic engaged in social activities. The ongoing presence of transmission pairs between 0–19 and 30–64 age groups can be attributed to interactions in parent-child and teacher-student relationships. Additionally, an observed surge in infections originating from the 0–19 age group during Period 4 is noteworthy.

Similarly, the proportion of diagonal elements in the regional group is high, with a notable prevalence of transmission pairs in the metropolitan area. This phenomenon is attributed to the high population density in the metropolitan area, serving as a hub for transportation and culture. In Period 1, the higher number of infections in Gyeongbuk+ compared to other regions is due to group infections in closed-off churches. The initial low incidence of infections in other regions is a result of limited population movement due to epidemic control policies.

**Figure 4:**
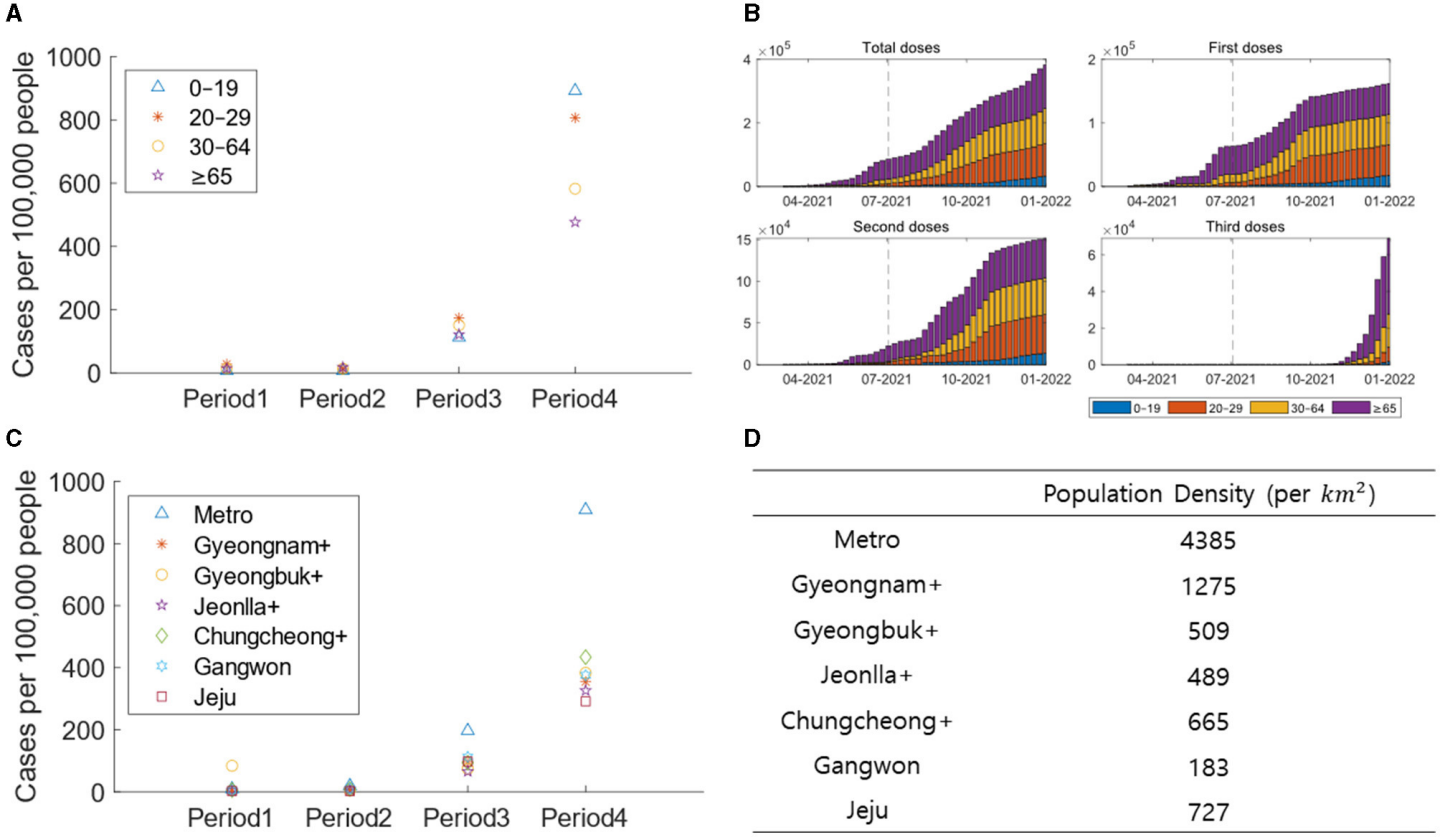
**(A)** The confirmed cases are displayed per 100,000 people based on age groups for each period. **(B)** The vaccine recipients are displayed per 100,000 people for each age group. **(C)** The confirmed cases are displayed per 100,000 people based on regional groups for each period. **(D)** The population density are displayed based on regional groups.

Figure 4 depicts demographic aspects, including age and region-specific confirmed cases, vaccination rates, etc. Figure 4A illustrates the number of confirmed cases per 100,000 people based on age and period. Age groups 0–19, 20–29, 30–64, and ≥65 are represented by blue triangles, red stars, yellow circles, and purple pentagrams, respectively. Figure 4B presents the weekly vaccination counts per 100,000 people for each age group in a stacked bar chart. Age groups 0–19, 20–29, 30–64, and ≥65 are represented by blue, red, yellow, and purple bars, respectively. Figure 4C displays the number of confirmed cases per 100,000 people based on regional groups for each period. Metro, Gyeongnam+, Gyeongbuk+, Jeonlla+, Chungcheong+, Gangwon, and Jeju are represented by blue triangles, red stars, yellow circles, purple pentagrams, green diamonds, sky-blue hexagrams, and violet squares, respectively. Figure 4D represents the population density for each regional group, calculated by dividing the population of the corresponding region by its area (in *km*^2^).

**Figure 5:**
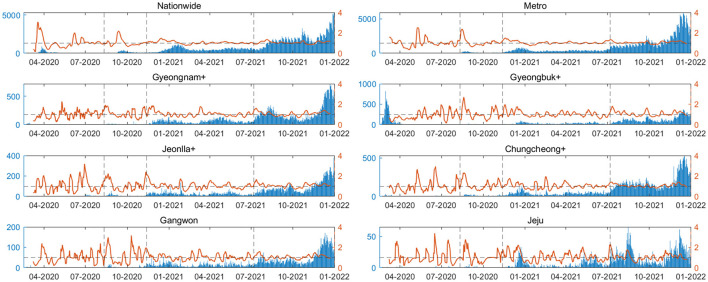
The time-varying reproduction number ($R_{t}$) is calculated for each regional group. The red line represents the reproduction number over time, while the blue bars indicate the confirmed cases for the respective dates. The dashed black line denotes the division between time periods.

Figure 5 presents the time-varying reproduction number ($R_{t}$) calculated for each regional group. The red line represents the reproduction number over time, while the blue bars indicate the confirmed cases for the respective dates. The dashed black line denotes the division between time periods. Using regional serial interval data for each periods, $R_{t}$ is calculated. The serial interval distribution is fitted to various distributions, with the optimal distribution determined through AIC analysis. This approach allows for a more accurate estimation of $R_{t}$. Different regions exhibit varying patterns in confirmed cases and $R_{t}$ values. In the metropolitan area, $R_{t}$ stabilizes close to 1 from the latter part of Period 2 onwards.

## 4 Discussion

We examined the changes in regional and age-specific characteristics over time using contact tracing data. Our study was intended to shed light on various strategies, including regional social distancing measures, school closure policies, and vaccinations. A key element of our research involved obtaining detailed information on transmission pairs, including the timing of symptom onset for the infector and infectee. This was made possible through the diligent efforts of Korean epidemiological investigators. Serial interval analysis has been a subject of research on both domestic and international fronts. In contrast to previous studies that relied on a limited dataset, our research stands out for its strength, drawing from an extensive dataset of 72,423 pairs (20, 21). This large dataset allows us to comprehensively explore and consider heterogeneity in a more robust manner.

Our study emphasizes the use of the serial interval to assess heterogeneity and evaluate public health measures such as non-pharmaceutical interventions and vaccine effectiveness. Additionally, we analyze sociodemographic factors based on age and region. Hong et al. (22) concludes that the serial interval increases with older age and a higher proportion of females. It underscores the importance of research revealing heterogeneity by gender and age, and highlights the need for further studies due to uncertainties in data collected during the pandemic. Shim et al. (23) confirms heterogeneity through epidemiological distributions, encompassing intervals from COVID-19 symptom onset to diagnosis, reporting, and death, considering region, age, gender, and period. It acknowledges limitations in not considering vaccination and underscores the necessity to reflect changes in social distancing and vaccination levels due to various public health policies. Ryu et al. (24) analyzes age-specific serial intervals, cluster types, and estimated $R_{t}$ to compare the characteristics of two epidemic waves. It acknowledges a limitation in not considering regional heterogeneity over time. Ryu et al. (25) estimates COVID-19 serial intervals, reproduction numbers, and superspreading potential during the dominance of the Delta variant in South Korea. It recognizes the limitation of not considering vaccination. Many studies emphasize the importance of researching heterogeneity, citing limitations such as small sample sizes and the failure to consider demographic public health measures and vaccination. Our study addresses these limitations by considering spatiotemporal heterogeneity in evaluating sociodemographic public health measures.

There are several strategies for effectively leveraging processed information concerning measures such as the serial interval and $R_{t}$ in epidemiological analysis and decision-making. Real-Time Monitoring emerges as a pivotal strategy, enabling the prompt identification of emerging trends and the timely adjustment of public health measures. For example, Forsberg White and Pagano (26) proposed a method for simultaneously estimating the basic reproductive number, $R_{0}$, and the serial interval for infectious disease epidemics using readily available surveillance data. These estimates can be acquired in real-time, empowering public health authorities to respond promptly. Another valuable strategy is scenario planning. By considering various scenarios regarding the transmission dynamics of the disease and calculating $R_{t}$ and the serial interval for each scenario, decision-makers can more effectively tailor interventions to suit the prevailing situation. Numerous studies have employed screening modeling to prevent the spread of diseases (27–29). For instance, Rajendrakumar et al. (30) utilized serial intervals to estimate the time-varying reproductive number and emphasized the importance of resuming travel gradually, while also advocating for the utilization of efficient tools such as vaccines and medications. By integrating these diverse strategies, health authorities can optimize the utilization of information such as the serial interval and $R_{t}$ to make evidence-based decisions, thereby effectively controlling the spread of COVID-19.

The mean serial intervals for 0–19 age group in Period 1–2 are 3.8 and 2.3 days, respectively (see to Supplementary Table S3). Compared to the overall serial interval mean, these values exhibit the largest differences in the mean serial interval by period and age, with variances of 0.8 and 0.7. This difference is attributed to the influence of negative serial intervals, allowing inference into the impact of presymptomatic transmission. In Periods 1 and 2, the respective proportions of negative serial intervals for this age group are 9.6% and 20.6%. While presymptomatic transmission was minimal in Period 1, a significant increase is observed from Period 2 onwards. In Period 1, Gyeongbuk+ shows a 8.1% higher proportion of negative serial intervals than the average, likely due to incomplete epidemiological investigations stemming from uncooperative religious characteristics. Over time, there is a trend toward the distribution of serial intervals and the proportion of negative serial intervals aligning with the overall mean across age and regional groups. This suggests that over time, public health measures have been effectively implemented in different age and regional groups.

Regarding Figure 3A, the prevalence of transmission pairs between individuals aged 0–19 and 30–64 highlights active transmission within families and school settings. Notably, the substantial representation of transmission pairs in the 30–64 age group across all periods can be attributed to the extensive economic activities within this age range, emphasizing the role of workplace-related infections in the spread of COVID-19. Additionally, the older adult population aged 65 and above constitutes a significant portion of the transmission pairs due to interactions within nursing homes, long-term care facilities, and the sheer size of this age group. Figure 3B underscores the significant influence of the metropolitan area, driven by high population density resulting from its role as a transportation, economic, and cultural hub. This heightened density contributes to increased disease transmission due to numerous social interactions. The elevated number of transmission pairs in Gyeongbuk+ during Period 1 can be attributed to a cluster outbreak in Shincheonji. The limited number of transmission pairs in regions outside the metropolitan area from Period 1 to Period 2 can be attributed to strict early social distancing measures that restricted inter-regional movement.

In contrast to Periods 1–3, there is a notable surge in the number of confirmed cases per 100,000 people among those aged 0–19 in Period 4. This disparity is evident in the differences in vaccination rates across age groups. In Period 4, the ≥65 age group, showing the lowest proportion of confirmed cases, also exhibits an increase in the rate of third-dose vaccinations. On the other hand, the vaccination rate for the 0–19 age group is significantly lower than other age groups, indicating the impact of vaccination. In Period 1, Gyeongbuk+ had the highest number of confirmed cases per 100,000 people due to a superspreading event. Subsequently, Metro, with the highest population density, witnesses the highest number of confirmed cases. This suggests that population density influences the pattern of transmission.

Through our research, we were able to analyze a dataset for transmission pairs based on time, allowing for an evaluation of epidemic prevention policies by region and age. The increased spread in the 0–19 age group during Period 4, unlike other periods, is attributed to changes in the school closure policies. Figure 1C indicates that both metropolitan and non-metropolitan areas uniformly implemented full in-person classes in November 2021. This resulted not only in an increase in infections among individuals aged 0–19 but also a significant rise in infections between 0–19 and 30–64 age groups. Epidemic prevention policies due to school closure policies in Periods 1–3 were effective. Additionally, except for Period 1, the metropolitan area consistently had a higher number of confirmed cases. The high number of transmission pairs suggests it as the most severely affected region. However, the actual implementation of proactive social distancing measures, as seen in Figure 1A, delayed the spread. This is evident in the $R_{t}$ displayed in Figure 5.

In Figure 5, the Metro area demonstrates $R_{t}$ stabilizing near 1 during the latter part of Period 2, indicating effective implementation of containment policies compared to other regions. However, most other regions experience a peak in $R_{t}$ until Period 4. These findings shed light on diverse regional transmission patterns and the varying impacts of containment policies, underscoring the importance of research that considers age and regional heterogeneity. Additionally, they provide insights into the evolving characteristics of the pandemic over time. By introducing parameters such as serial interval and $R_{t}$, we provide a fresh perspective on epidemic prevention policies and non-pharmaceutical interventions, going beyond simple figures like the number of confirmed cases and deaths.

Our study comes with several limitations. We conducted our analysis based on parameters that could be empirically derived from available data, without accounting for various other parameters. The use of the infector as a criterion based on region and age could introduce bias into our analysis. Other studies, such as those by Lau et al. (31), Hart et al. (32), and Xin et al. (33), have approached this research question from diverse angles, estimating infection-related parameters like the generation interval and latent period. The choice of infector as the basis for our study was influenced by its strong representation of behavioral patterns, as shown in Kim et al. (13). In our future work, we plan to introduce additional parameters that can be assessed from various perspectives. Also, establishing criteria based on both the infector and the infectee and exploring differences from multiple angles will provide a solid foundation for gaining new insights in this field.

Our study presents gender proportions based solely on the data without exploring gender differences across various parameter perspectives. Upon analyzing the proportions within the dataset, no significant gender disparities were noted. However, in future investigations, we plan to estimate parameters while taking into account distinctions not only across regions and age groups but also between genders, with the goal of investigating potential heterogeneity. Lastly, our study is specifically focused on estimating $R_{t}$ without integrating mobility (34–36). In a forthcoming study, we will examine the influence of mobility on $R_{t}$ (37–40). Our study primarily assesses changes in key epidemiological parameters of COVID-19 over time, taking into account the variations by region and age in South Korea. Through this analysis, we provide an evaluation of control policies and measures, emphasizing the significance of research that accounts for this heterogeneity.

## Data availability statement

The data analyzed in this study is subject to the following licenses/restrictions: the original contributions presented in the study are included in the article material, further inquiries can be directed to the corresponding author. Requests to access these datasets should be directed to SL, sunmilee@khu.ac.kr.

## Author contributions

HL: Conceptualization, Formal analysis, Methodology, Visualization, Writing – original draft. GL: Conceptualization, Formal analysis, Writing – review & editing. TK: Conceptualization, Formal analysis, Methodology, Visualization, Writing – original draft. SK: Investigation, Visualization, Writing – original draft. HK: Investigation, Visualization, Writing – original draft. SL: Conceptualization, Formal analysis, Funding acquisition, Supervision, Writing – original draft, Writing – review & editing.
