# Supplementary material for: Variability in the serial interval of COVID-19 in South Korea: a comprehensive analysis of age and regional influences
Source: Front Public Health. 2024 Mar 7;12:1362909. doi: 10.3389/fpubh.2024.1362909 (PMC10955094; doi:10.3389/fpubh.2024.1362909)
Supplement: Supplementary file 1 [file Data_Sheet_1.PDF]

Variability in the Serial Interval of COVID-19 in South Korea: A Comprehensive Analysis of Age and Regional Influences

1 SUPPLEMENTARY FIGURES AND TABLES

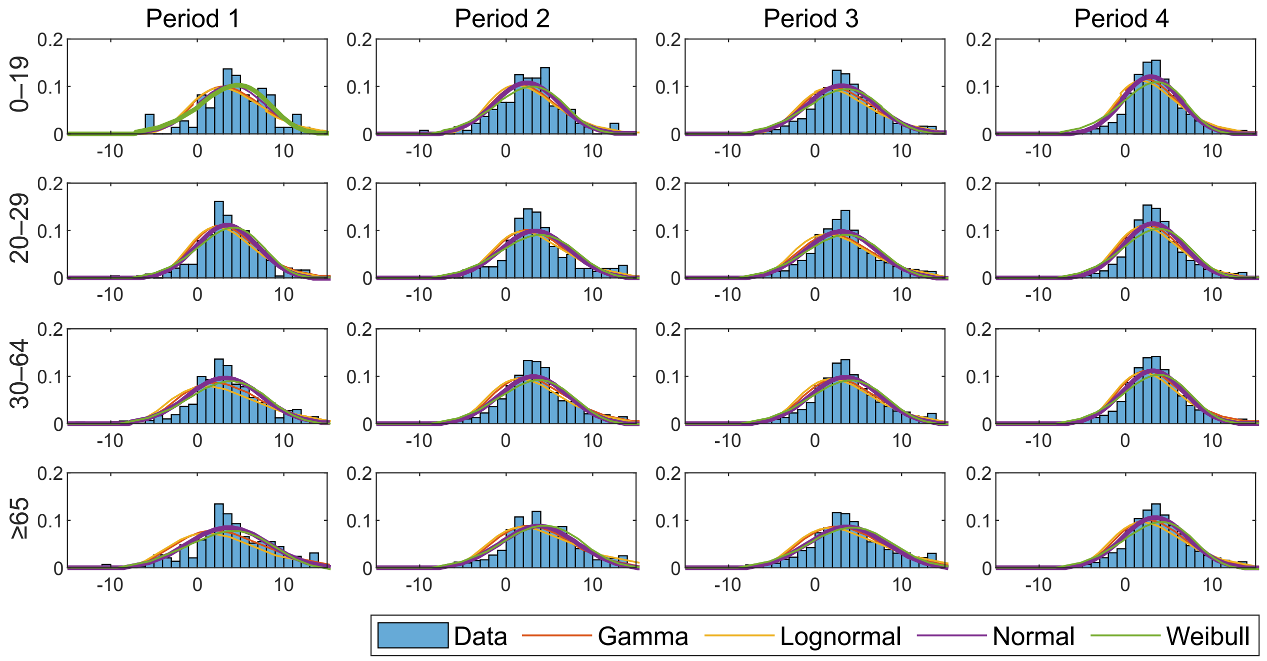

Figure S1. The distributions of the serial interval are displayed based on age groups for each period. Each distribution is fitted with gamma, lognormal, normal, and Weibull. The best-fitted distributions are represented by the bold line.

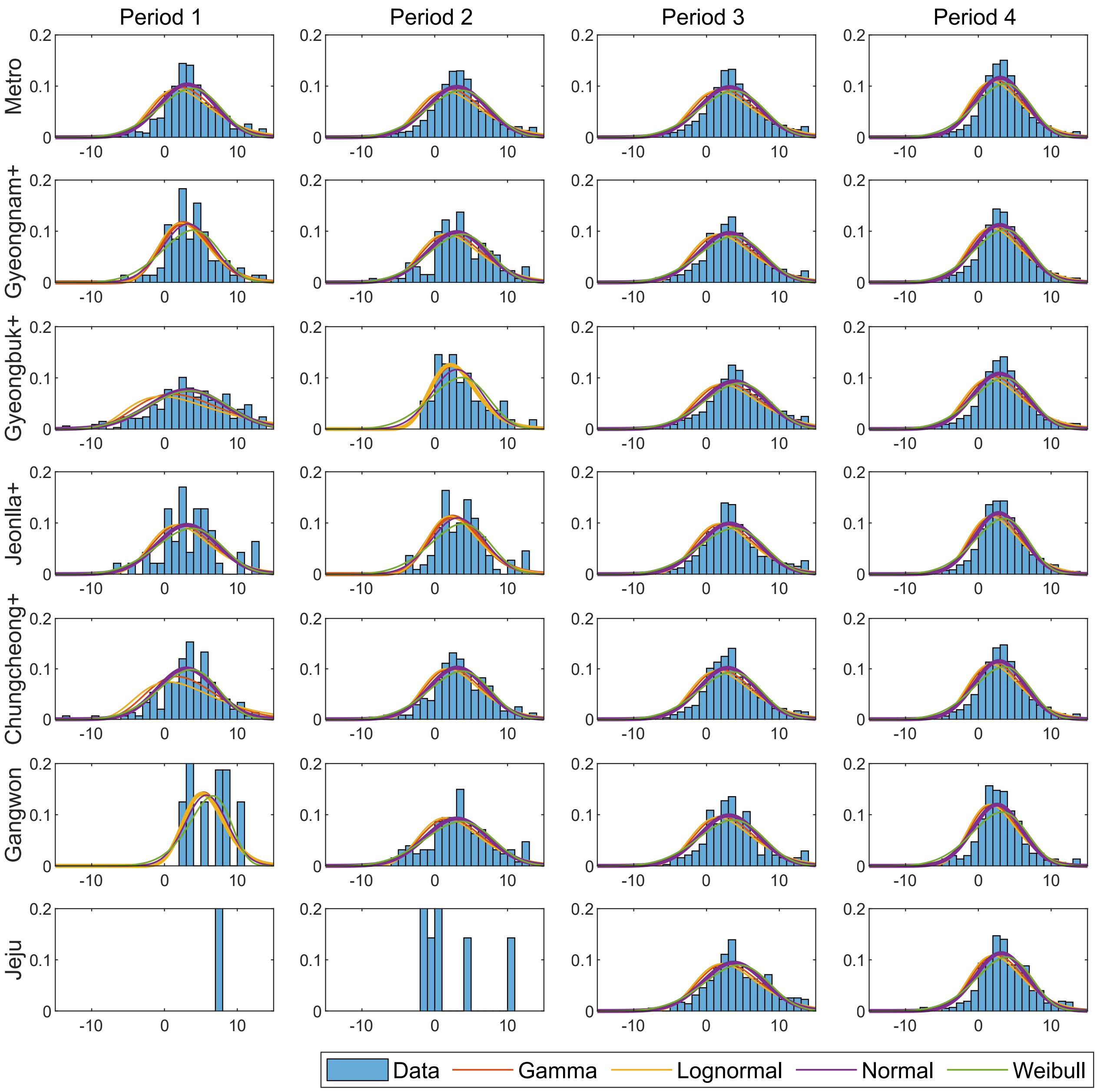

Figure S2. The distributions of the serial interval are displayed based on region groups for each period. Each distribution is fitted with gamma, lognormal, normal, and Weibull. The best-fitted distributions are represented by the bold line.

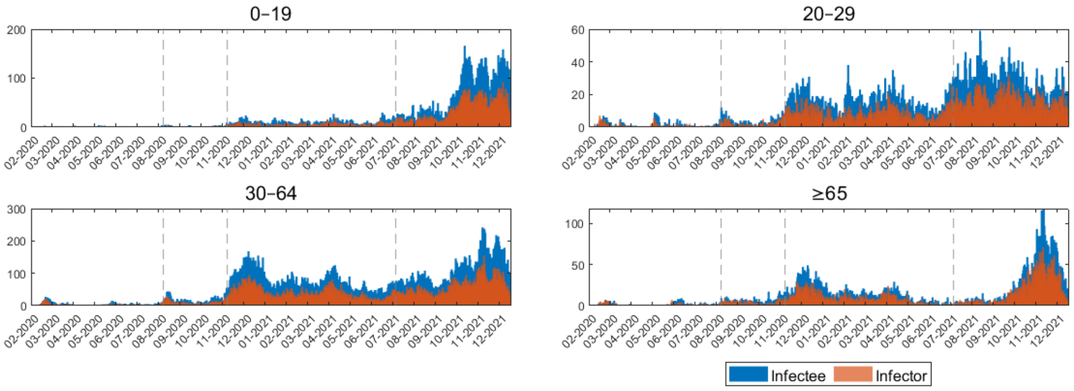

Figure S3. Daily number of symptomatic individuals with symptoms for infectors and infectees are shown based on age. Daily symptomatic cases for both infectors and infectees is represented by orange and blue bars, respectively.

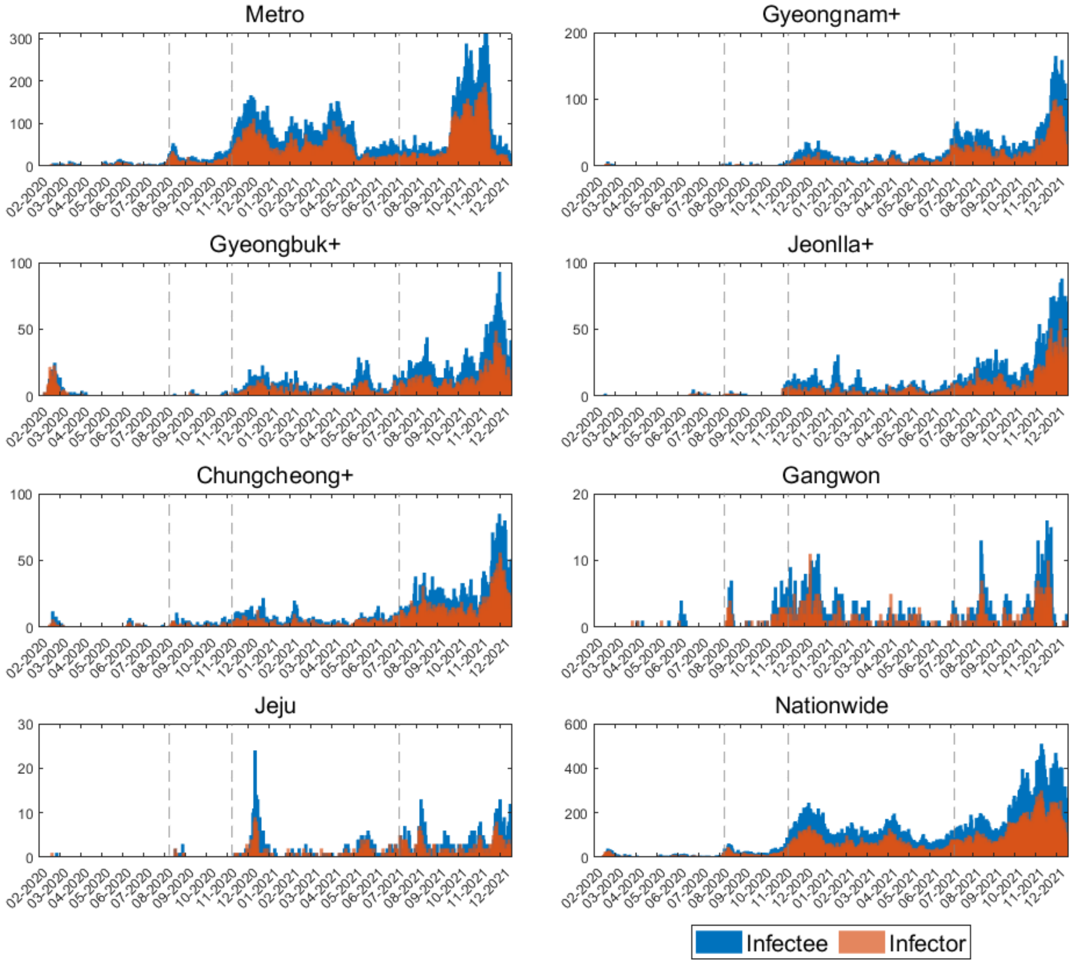

Figure S4. Daily number of symptomatic individuals with symptoms for infectors and infectees are shown based on region. Daily symptomatic cases for both infectors and infectees is represented by orange and blue bars, respectively.

		**Period 1**	**Period 2**	**Period 3**	**Period 4**
0–19	Male	59 (79%)	106 (75%)	1491 (62%)	7189 (59%)
	Female	16 (21%)	36 (25%)	901 (38%)	4896 (41%)
20–29	Male	167 (69%)	177 (54%)	2056 (57%)	2858 (57%)
	Female	74 (31%)	148 (46%)	1525 (43%)	2184 (43%)
30–64	Male	355 (37%)	817 (45%)	9268 (49%)	10022 (51%)
	Female	602 (63%)	979 (55%)	9541 (51%)	9564 (49%)
≥ 65	Male	141 (46%)	289 (46%)	1910 (51%)	2653 (52%)
	Female	168 (54%)	346 (54%)	1847 (49%)	2408 (48%)
Metro	Male	455 (52%)	1062 (49%)	10373 (52%)	9894 (57%)
	Female	426 (48%)	1120 (51%)	9520 (48%)	7342 (43%)
Gyeongnam+	Male	43 (59%)	85 (63%)	1413 (47%)	4606 (52%)
	Female	30 (41%)	51 (37%)	1567 (53%)	4261 (48%)
Gyeongbuk+	Male	134 (35%)	20 (35%)	950 (52%)	2319 (51%)
	Female	248 (65%)	37 (65%)	883 (48%)	2232 (49%)
Jeonlla+	Male	20 (41%)	47 (42%)	826 (51%)	2597 (52%)
	Female	29 (59%)	64 (58%)	788 (49%)	2423 (48%)
Chungcheong+	Male	50 (32%)	96 (39%)	699 (51%)	2739 (55%)
	Female	106 (68%)	148 (61%)	660 (49%)	2201 (45%)
Gangwon	Male	2 (13%)	53 (40%)	206 (53%)	223 (47%)
	Female	14 (87%)	78 (60%)	183 (47%)	251 (53%)
Jeju	Male	1 (100%)	5 (71%)	182 (51%)	301 (51%)
	Female	0 (0%)	2 (29%)	175 (49%)	288 (49%)

Table S1: The number of genders is represented by infector over given periods.

		**Period 1**	**Period 2**	**Period 3**	**Period 4**
0–19	Male	59 (79%)	106 (75%)	1491 (62%)	7189 (59%)
	Female	16 (21%)	36 (25%)	901 (38%)	4896 (41%)
20–29	Male	167 (69%)	177 (54%)	2056 (57%)	2858 (57%)
	Female	74 (31%)	148 (46%)	1525 (43%)	2184 (43%)
30–64	Male	355 (37%)	817 (45%)	9268 (49%)	10022 (51%)
	Female	602 (63%)	979 (55%)	9541 (51%)	9564 (49%)
≥ 65	Male	141 (46%)	289 (46%)	1910 (51%)	2653 (52%)
	Female	168 (54%)	346 (54%)	1847 (49%)	2408 (48%)
Metro	Male	455 (52%)	1062 (49%)	10373 (52%)	9894 (57%)
	Female	426 (48%)	1120 (51%)	9520 (48%)	7342 (43%)
Gyeongnam+	Male	43 (59%)	85 (63%)	1413 (47%)	4606 (52%)
	Female	30 (41%)	51 (37%)	1567 (53%)	4261 (48%)
Gyeongbuk+	Male	134 (35%)	20 (35%)	950 (52%)	2319 (51%)
	Female	248 (65%)	37 (65%)	883 (48%)	2232 (49%)
Jeonlla+	Male	20 (41%)	47 (42%)	826 (51%)	2597 (52%)
	Female	29 (59%)	64 (58%)	788 (49%)	2423 (48%)
Chungcheong+	Male	50 (32%)	96 (39%)	699 (51%)	2739 (55%)
	Female	106 (68%)	148 (61%)	660 (49%)	2201 (45%)
Gangwon	Male	2 (13%)	53 (40%)	206 (53%)	223 (47%)
	Female	14 (87%)	78 (60%)	183 (47%)	251 (53%)
Jeju	Male	1 (100%)	5 (71%)	182 (51%)	301 (51%)
	Female	0 (0%)	2 (29%)	175 (49%)	288 (49%)

Table S2: The number of genders is represented by infectee over given periods.

		Period 1	Period 2	Period 3	Period 4
0-19	Mean	3.8	2.3	3	2.9
	Std	3.9	3.7	3.9	3.3
20-29	Mean	3.2	3.1	2.8	2.9
	Std	3.5	3.9	4	3.4
30-64	Mean	3	2.9	3.2	2.9
	Std	4.2	3.9	4	3.5
≥ 65	Mean	3.3	3.5	3.5	3.2
	Std	4.7	4.3	4.5	3.7
Metro	Mean	3.1	3	3.1	3
	Std	3.9	4	4.1	3.4
Gyeongnam+	Mean	3.2	3	3.1	2.9
	Std	3.5	4.1	4.1	3.5
Gyeongbuk+	Mean	3.1	3	3.8	2.9
	Std	5.2	3.4	4.3	3.7
Jeonlla+	Mean	3.1	3.2	3.2	2.8
	Std	4.2	3.6	4	3.3
Chungcheong+	Mean	3	3.1	2.9	2.9
	Std	4	3.9	3.9	3.5
Gangwon	Mean	-	3	3.1	2.6
	Std	-	4.3	4	3.3
Jeju	Mean	-	-	3.7	3.1
	Std	-	-	4.2	3.5

Table S3: The statistics for the mean and standard deviation (std) of the serial interval based on age and regional groups for each period.

	Period 1	Period 2	Period 3	Period 4
0-19	Weibull	Normal	Normal	Normal
20-29	Normal	Normal	Normal	Normal
30-64	Normal	Normal	Normal	Normal
≥ 65	Normal	Normal	Normal	Normal
Metro	Normal	Normal	Normal	Normal
Gyeongnam+	Gamma	Normal	Normal	Normal
Gyeongbuk+	Normal	Lognormal	Normal	Normal
Jeonlla+	Normal	Gamma	Normal	Normal
Chungcheong+	Normal	Normal	Normal	Normal
Gangwon	-	Normal	Normal	Normal
Jeju	-	-	Normal	Normal

Table S4: The best fitted distribution of age and region-specific transmission pairs by periods

		Period 1	Period 2	Period 3	Period 4
0-19	Gamma	415	754	13154	63495
	Lognormal	420	765	13388	64811
	Normal	408	746	12975	62515
	Weibull	408	753	13091	63502
20-29	Gamma	1314	1692	19928	26419
	Lognormal	1332	1708	20357	26905
	Normal	1297	1689	19605	26141
	Weibull	1309	1712	19768	26624
30-64	Gamma	5351	9804	101732	103528
	Lognormal	5576	10017	103698	105066
	Normal	5160	9642	100388	102488
	Weibull	5205	9728	101311	103897
≥ 65	Gamma	1773	3504	21172	27390
	Lognormal	1825	3572	21574	28103
	Normal	1722	3443	20858	26808
	Weibull	1727	3458	20955	27061
Metro	Gamma	4821	11884	108142	90531
	Lognormal	4941	12153	110498	92251
	Normal	4724	11671	106435	89328
	Weibull	4773	11770	107373	90782
Gyeongnam+	Gamma	381	750	16557	47521
	Lognormal	383	760	16765	48298
	Normal	382	742	16415	46977
	Weibull	389	744	16526	47535
Gyeongbuk+	Gamma	2158	290	10191	24506
	Lognormal	2247	288	10366	25084
	Normal	2076	295	10028	24029
	Weibull	2083	302	10056	24298
Jeonlla+	Gamma	271	596	8794	26314
	Lognormal	273	597	8877	26766
	Normal	270	599	8777	25989
	Weibull	272	609	8879	26359
Chungcheong+	Gamma	896	1362	7420	26164
	Lognormal	951	1375	7555	26633
	Normal	842	1354	7323	25891
	Weibull	848	1364	7398	26294
Gangwon	Gamma	-	735	2145	2349
	Lognormal	-	739	2178	2362
	Normal	-	735	2125	2348
	Weibull	-	738	2150	2396
Jeju	Gamma	-	-	2032	3137
	Lognormal	-	-	2053	3177
	Normal	-	-	2017	3102
	Weibull	-	-	2024	3131

Table S5: The AIC (Akaike Information Criterion) values for gamma, lognormal, normal, and Weibull distributions fitted to the serial interval data based on age and regional groups for each period.
